# Spirometry Reference Equations for Central European Populations from School Age to Old Age

**DOI:** 10.1371/journal.pone.0052619

**Published:** 2013-01-08

**Authors:** Mascha K. Rochat, Ruediger P. Laubender, Daniela Kuster, Otto Braendli, Alexander Moeller, Ulrich Mansmann, Erika von Mutius, Johannes Wildhaber

**Affiliations:** 1 Children's Hospital, University of Munich, Munich, Germany; 2 Institute for Medical Informatics, Biometry und Epidemiology, University of Munich, Munich, Germany; 3 Division of Respiratory Medicine, Children's Hospital, University of Zurich, Zurich, Switzerland; 4 Zurich Lung Association, Zurich, Switzerland; 5 Department of Paediatrics, Hospital of Fribourg and Faculty of Medicine, University of Fribourg, Fribourg, Switzerland; 6 Department of Pediatrics, Lausanne University Hospital, Lausanne, Switzerland; University of California San Francisco, United States of America

## Abstract

**Background:**

Spirometry reference values are important for the interpretation of spirometry results. Reference values should be updated regularly, derived from a population as similar to the population for which they are to be used and span across all ages. Such spirometry reference equations are currently lacking for central European populations.

**Objective:**

To develop spirometry reference equations for central European populations between 8 and 90 years of age.

**Materials:**

We used data collected between January 1993 and December 2010 from a central European population. The data was modelled using “Generalized Additive Models for Location, Scale and Shape” (GAMLSS).

**Results:**

The spirometry reference equations were derived from 118'891 individuals consisting of 60'624 (51%) females and 58'267 (49%) males. Altogether, there were 18'211 (15.3%) children under the age of 18 years.

**Conclusion:**

We developed spirometry reference equations for a central European population between 8 and 90 years of age that can be implemented in a wide range of clinical settings.

## Introduction

“Spirometry measurements are important in diagnosis and follow-up of patients with respiratory diseases and their interpretation relies on the availability and use of appropriate reference equations [1]. In Europe, the most commonly used reference equations are outdated [2] and the continued publication of new reference equations [3] reflects the widespread recognition of the limitations of the existing ones. Most reference equations are indeed inappropriate for central European populations as they have either been derived from a small or non-European population [3] or used statistical methods that cannot adequately model the complexity of age-dependent lung function [2]”. Additionally, published reference values are mostly derived from healthy never-smoking populations of restricted age ranges [3] and should not be extrapolated beyond the published range [1], [4], [5]. Practically, however, clinicians often track disease progression over long periods or assess effectiveness of therapy over time in patients who are not “healthy never-smokers”. There is, therefore, an important need for practical reference values spanning across all ages derived from a population most similar to that for which the equations are to be used.

Such reference equations are statistically challenging as on the one hand individual spirometry measurements are determined by age, sex, height, health status, ethnicity, equipment and general population characteristics (so called “cohort effect”) [1], [4], [6] and the European Respiratory Society (ERS)/American Thoracic Society (ATS) recommend taking these characteristics into account when developing and updating reference equations [1]. On the other hand, the lung volume changes according to height and age with a skewed distribution [7], [8]. Statistical methods taking multiple variables as well as this complex distribution into account have been developed and compared [9] in recent years. A possible approach that has been applied to spirometry data are Generalized Additive Models for Location, Scale and Shape (GAMLSS) methods. GAMLSS allows modelling of data with skewed and kurtotic distribution and is therefore ideal for spirometry reference equations including transition from childhood to adulthood [10], [11].

The aim of this study was to develop reference equations for a central European Population between 8 and 90 year olds.

## Materials and Methods

In this study we used data collected by the “LuftiBus” which is a project that has been described in detail previously [12], [13]. Briefly, the “LuftiBus” is a mobile bus equipped with two flow-sensing spirometers that tours the greater Zurich (Switzerland) area and offers spirometry measurements to the general population. Spirometry data were recorded electronically along with data from a standardised interviewer-administered questionnaire collecting basic information on health and lifestyle of the subjects. Lung function tests were charged 10 CHF for adults and 5 CHF for children if the bus was not leased by an organisation or a community in which case the test was free of charge. When the bus was leased by schools, entire classrooms were tested. In children, weight (kg) and standing height (cm) were measured according to WHO recommendations [14], in adults they were either asked or measured.

### Study Population

For this analysis we used the data collected from volunteers between January 1993 and December 2010. In the course of the years the “LuftiBus” visited each village of the Zurich County. In each village a similar proportion of the population was tested. This proportion ranged from 0.66% in Andelfingen to 2.05% in Dielsdorf. Additionally, the age distribution of the “LuftiBus” dataset is similar to the age distribution of the Swiss population with the exception for an over-representation of teenagers [15]. Although the population tested was mainly of Western European descent, ethnicity was recorded as of 2004 (33.7% of the whole population). Non-Western European descent participants accounted for 375 (2.04%) men and 355 (1.98%) women and were excluded from the analysis. They were the only individuals excluded from the dataset. The Zurich population is representative of Central and Western European populations [16], or North-West/Central European populations [17].

### Spirometry

The “LuftiBus” is equipped with two computerised pneumotachographs (SensorMedics1 Vmax Legacy 20c spirometer run by Vision 7-2b software; VIASYS, Yorba Linda, CA, USA). The volume signal of the equipment was calibrated at least once daily with a 3-L syringe. Tests were performed in a sitting position according to American Thoracic Society (ATS) guidelines until end of 2005 and ATS/European Respiratory Society (ERS) guidelines as of 2006 without nose-clips and after oral instruction by the technician [18], [19]. Participants were assisted by trained spirometry technicians who performed immediate on-screen evaluation of major acceptability criteria (including start, duration and end of test) in addition to the automated review performed by the computer software. As recommended by the ATS/ERS task force [19] subjects were asked to perform up to a maximum of eight manoeuvres in an attempt to obtain reproducible results. The largest forced vital capacity (FVC) and forced expiratory volume in one second (FEV1) were selected. All other parameters [FEV1/FVC ratio, peak expiratory flow (PEF), mean expiratory flow at 75%, 50%, 25% of expired volume (MEF_75_, _50_, _25_)] were taken from the trial with the largest sum of FVC and FEV1.

### Definition of variables

For the analysis we defined the two exploratory variables “smoking” and “sick”. Smoking was defined as a cumulative self-reported smoking history of more than one pack-year. A pack-year being defined as years of smoking times the number of cigarettes smoked per day divided by 20. For the exploratory variable “smoking” passive smokers were considered non-smokers. Sick volunteers were defined as meeting one of the following criteria: i) common cold at the time of the measurement or ii) lung disease at the time of the measurement, which included acute bronchitis or respiratory symptoms (cough, wheezing, phlegm, shortness of breath during rest or exertion); asthma medication at the time of the measurement; history of asthma; history of chronic obstructive pulmonary disease; chronic bronchitis or a history of other lung diseases (e.g. lung surgery, pulmonary embolism). Volunteers with non-respiratory diseases such as diabetes or heart diseases were included in the healthy group. For the analysis we defined 4 health groups: healthy/non-smoker, healthy/smoker, sick/non-smoker and sick/smoker.

### Statistical analysis

Statistical analysis was performed with the statistical software “R” version 2.13.1 (R Development Core Team 2011) with the packages ‘gamlss’ (version 4.0-8) and ‘gamlss.tr’ (version 4.0-4) for the GAMLSS models [10], [11], [20] and with the package ‘quantreg’ (version 4.71) for the quantile regression models [21]. Within the GAMLSS framework we used the four-parametric Box-Cox power exponential density distribution function (BCPE(**μ**, **σ**, **ν**, **τ**)) as this distribution allows modelling of the expectation (**μ**), the variance (**σ**), the skewness (**ν**) as well as the kurtosis (**τ**) [10] and a truncated BCPE distribution for FEV1/FVC as that endpoint cannot exceed 100%. Due to the non-linear relation between the spirometry parameters and age we used a bent hyperbola model for the **μ** link with two change points and two transition smoothness parameters. Further, the non-linear relation between the spirometry parameters and age for the **σ** link was modelled by fractional polynomials of the 2^nd^ degree. The change points and the transition smoothness parameters were estimated using the L-BFGS-B algorithm and within the GAMLSS models framework using the generalized Akaike's information criteria (GAIC) with a penalty of 3 and Bayesian Information Criterion (BIC). Continuous variables are presented as median and inter-quartile range. We modelled the relation between the spirometry parameters and the covariates age, height, sex, smoking status and disease status. Besides, several models with interaction terms formed of the variables age, sex and height were fitted and selected using GAIC with a penalty 3 and BIC.

## Results

### Study population

**Figure 1:**
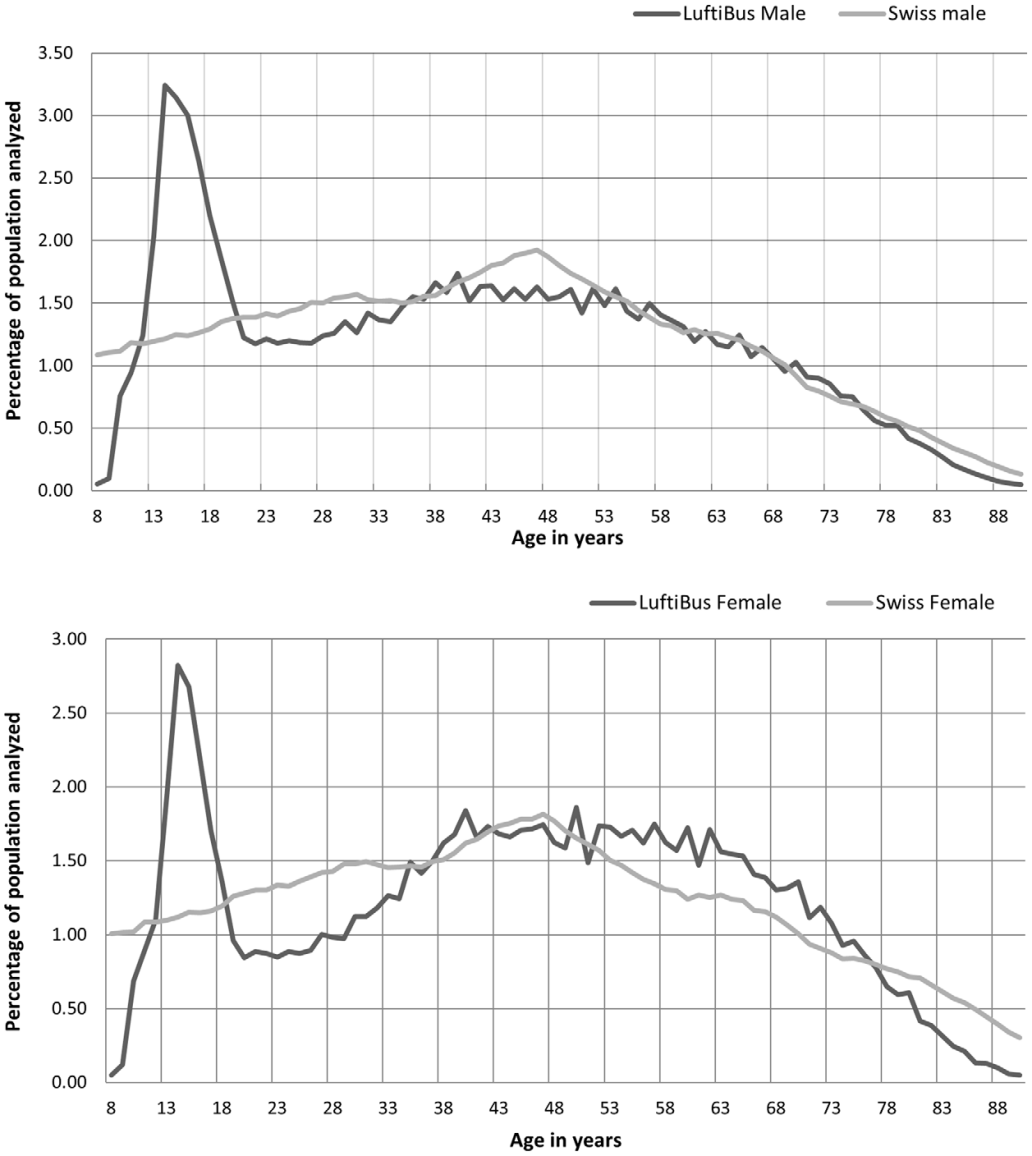
Age distribution of the reference population. A comparison with the age distribution of the Swiss population in 2011 is made.

From a total of 128'568 measurements 9'677 were excluded due to age (<8 years, >90 years) incomplete data or non-Western-European origin. The spirometry reference equations were derived from 118'891 individuals consisting of 60'624 (51%) females and 58'267 (49%) males. In total there were 18'211 (15.3%) children under the age of 18 years. The age distribution of the study population is shown in Figure 1. The main characteristics of the study population can be taken from Table 1. In adults 58.9% of the women and 43.8% of the men were never smokers. All together 34.9% of the individuals under the age of 18 were either active (19.9%) or passive smokers (14.9%). Of all individuals, 66.3% where healthy, 6.8% had a common cold at the time of the measurement, 17.0% had a lung disease and 9.8% a non lung-related disease such as diabetes or heart disease.

**Table 1:** Characteristics of the study population.

N = 118'891	Female n = 60'624		Male n = 58'267	
	Adults	Children (<18)	Adults	Children (<18)
	52'245 (100%)	8'379 (100%)	48'435 (100%)	9'832 (100%)
**Age**	50 (25)	14 (3)	47 (27)	15 (3)
**Smoking:**				
**Never-Smokers**	30'753 (58.9%)	5'664 (67.6%)	21'207 (43.8%)	6'169 (62.7%)
**Smokers/Ex- Smokers**	18'419 (35.3%)	1'436 (17.1%)	25'769 (53.2%)	2'208 (22.5%)
**Passive smokers**	3'073 (5.9%)	1'279 (15.3%)	1'459 (3.0%)	1'455 (14.8%)
**Health status:**				
**Healthy**	33'256 (63.7%)	5'628 (67.2%)	33'211 (68,6%)	6'770 (68.9%)
**Common cold**	2'610 (5.0%)	1'134 (13.5%)	3'040 (6.3%)	1'263 (12.8%)
**Lung diseases**	9'745 (18.7%)	1'479 (17.7%)	7'378 (15.2%)	1'652 (16.8%)
**Other diseases**	6'634 (12.7%)	138 (1.6%)	4'806 (9.9%)	147 (1.5%)

For age we reported medians and inter-quartile range (in brackets) since the distribution was skewed.

Other diseases include all non-lung diseases such as diabetes, heart diseases, etc.

### Reference equation modelled with GAMLSS

The lung function parameters FEV1, FVC, PEF, MEF25, MEF50, MEF75 were modelled with the Box-Cox power exponential density distribution function (BCPE(**μ**, **σ**, **ν**, **τ**)). A truncated BCPE(**μ**, **σ**, **ν**, **τ**) function was used to model the lung function parameter FEV1/FVC. The BCPE distribution was necessary as it was not possible to renounce modelling the kurtosis (when using e.g. the BCCG distribution) as this would worsen the model fit and increases the BIC (GAIC) in the models for all endpoints. Residual analyses based on worm plot were done in order to identify model inadequacies and were performed graphically for all models (Figures S1 and S2). A good model fit was achieved as only about 1484 (1.21%) individuals were not on the QQ-line. The BCPE(**μ**, **σ**, **ν**, **τ**) function gives a distribution from which the 5th quantile can be predicted. This is the quantile generally recommended for the lower limit of the normal range. The reference values (5th quantile), according to the GAMLSS model, can be calculated by the four functions in Table 2 and transformed to z-scores as described by the formula 1 of reference [10] (Figure S3).

**Table 2:** GAMLSS reference equations.

Reference equation for the mean (μ):							
g_1_(μ) = β_0_+β_1_ *age+β_2_ * √ ((age−α_1_)^2^+γ_1_^2^)+β_3_ * √ ((age−α_2_)^2^+γ_2_^2^)+β_4_ *sex+β_5_ *height+β_6_ *smoker+β_7_ *sick+β_8_ * age * sex+β_9_ * √ ((age−α_1_)^2^+γ_1_^2^) * sex+β_10_ * √ ((age−α_2_)^2^+γ_2_^2^) * sex+β_11_ * height * sex							
	FEV1	FVC	FEV1/FVC	PEF	MEF25	MEF50	MEF75
Regression parameters (β)							
β_0_	−3.708	−5.593	105.386	−0.841	0.956	−0.627	−3.745
β_1_	0.044	0.025	0.516	0.154	0.098	0.097	0.071
β_2_	−0.068	−0.047	−0.919	−0.203	−0.148	−0.146	−0.118
β_3_	−0.013	−0.017	0.254	−0.088	−0.063	−0.017	0.046
β_4_	1.586	2.140	3.520	0.550	0.284	1.596	0.551
β_5_	0.043	0.061	−0.153	0.073	0.048	0.029	0.010
β_6_	−0.033	0.024	−1.443	−0.135	−0.048	−0.149	−0.126
β_7_	−0.086	−0.061	−1.028	−0.162	−0.322	−0.277	−0.091
β_8_	−0.024	−0.018	−0.083	−0.110	−0.074	−0.054	−0.028
β_9_	0.027	0.024	−0.088	0.119	0.088	0.063	0.029
β_10_	0.003	0.000	0.172	0.035	0.029	0.005	0.004
β_11_	−0.011	−0.015	−0.005	−0.013	−0.008	−0.009	−0.003
Changepoints (α)							
α_1_	18.789	22.080	15.964	19.688	19.436	17.194	16.505
α_2_	37.006	38.368	23.828	50.902	57.602	56.979	20.000
Transition smoothness (γ)							
γ_1_	0.789	0.587	0.285	1.067	1.494	0.965	0.100
γ_2_	12.143	9.122	6.771	27.527	17.050	3.257	74.096

Reference equation for the variance (σ):							
g_2_(σ) = exp[β_0_+β_1_ *(age ^p1^)+β_2_ *(age ^p2^)+β_3_ *height+β_4_ *sex+β_5_ *smoker+β_6_ *sick]							
	FEV1	FVC	FEV1/FVC	PEF	MEF25	MEF50	MEF75
Regression parameters (β)							
β_0_	−1.543	−1.483	−2.719	−0.690	−1.410	−0.991	−2.041
β_1_	0.269	0.276	−0.174	−0.162	0.056	0.014	−1.020
β_2_	0.012	0.011	0.025	0.011	0.001	−0.001	1.465
β_3_	−0.004	−0.004	0.002	−0.005	−0.001	−0.002	−0.003
β_4_	−0.095	−0.048	−0.009	−0.063	−0.148	−0.113	−0.026
β_5_	0.052	0.022	0.062	0.050	0.061	0.078	0.069
β_6_	0.132	0.069	0.094	0.069	0.128	0.118	0.078
Power for the fractional polynomials #							
p1	−1	−1	1	0.5	0	2	0
p2	2	2	2	2	3	3	0.5
# if p1≠p2 then β1*(age ^p1^)+β2*(age ^p2^); if p1 = p2 = p then β1*(age ^p^)+β2*(age ^p^)* ln(age);If p1 = 0 then use the natural logarithm (ln)							

Reference equation for the skewness (ν):							
g_3_(ν) = β_0_+β_1_ * age+β_2_ * height+β_3_ * sex+β_4_ * smoker+β_5_ * sick							
	FEV1	FVC	FEV1/FVC	PEF	MEF25	MEF50	MEF75
Regression parameters (β)							
β_0_	−0.130	−3.053	2.762	−0.854	0.291	0.946	0.618
β_1_	0.007	0.005	0.006	0.005	0.010	0.001	−0.005
β_2_	0.005	0.021	−0.007	0.009	0.001	−0.003	−0.001
β_3_	0.021	−0.093	−0.347	−0.206	−0.044	0.074	−0.022
β_4_	0.245	0.120	0.431	0.148	0.112	0.103	−0.003
β_5_	0.326	0.173	0.907	0.019	0.197	0.201	0.074

Reference equation for the kurtosis (τ):							
g_4_(τ) = exp[β_0_+β_1_ * age+β_2_ * height+β_3_ * sex+β_4_ * smoker+β_5_ * sick]							
	FEV1	FVC	FEV1/FVC	PEF	MEF25	MEF50	MEF75
Regression parameters (β)							
β_0_	0.166	−0.121	−0.046	0.528	0.106	0.280	0.603
β_1_	0.001	0.001	−0.001	0.001	0.001	0.001	0.002
β_2_	0.002	0.003	0.003	0.001	0.003	0.002	−0.001
β_3_	−0.014	−0.014	0.014	−0.066	−0.090	0.060	0.023
β_4_	0.015	0.007	0.015	0.022	0.038	0.003	0.018
β_5_	−0.026	−0.047	0.049	−0.039	−0.006	0.002	0.040

The variables are coded as followed:

Age: years; height: cm, sex: male = 0, female = 1; smoker: non-smoker = 0; smoker = 1*; sick: healthy = 0; sick = 1*.

* definition of smoker and sick can be found in the methods section of the paper.

**Note:** to compare a patient with a “healthy-non-smoker” population, “smoker” and “sick” must be set to “0” even if the patient is a smoker and has a pulmonary pathology.

### Comparison between the four health groups

**Figure 2:**
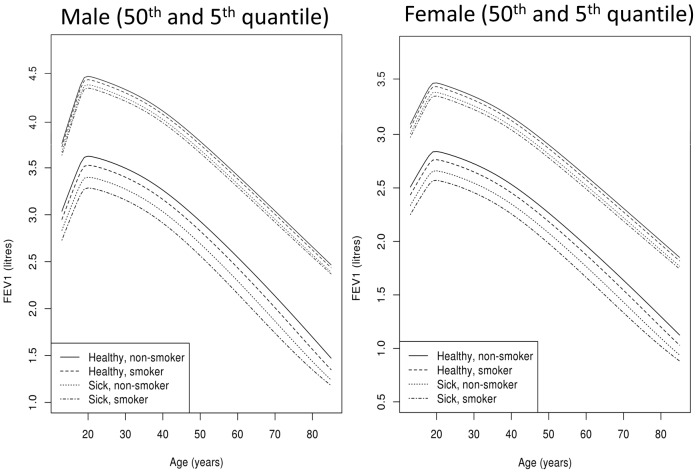
Comparison between the four health groups. The lung function parameter FEV1 is compared between the four health groups at ages between 8–90 years old. For this comparison only men of 175 cm and women of 165 cm were included. The 5^th^ quantile indicates the lower limit of normal for each group. FEV1: forced expiratory volume in one second. The four health groups are: healthy/non-smoker, healthy/smoker, sick/non-smoker and sick/smoker.

Our reference equations not only include information on age, sex and height but also on health and smoking status. This allows us to model the entire population and produce adaptable reference equations, where smokers can be compared to a smoking population. To illustrate this concept, Figure 2 shows a graphical representation of four different populations: “healthy/non-smoker” (54'488, 45.5%), “healthy/smoker” (36'760, 30.7%), “sick/non-smoker” (17'127, 14.3%) and “sick/smoker” (11'391, 9.5%). The biggest difference between these populations can be seen for the 5^th^ quantile which is generally used as the lower limit of normal. Not surprisingly, the individuals with the highest prediction values are the “healthy/non-smokers”. The “sick” individuals have the lowest values. A mean difference of 0.33 litres in men and 0.27 litres in women is seen between healthy/non-smokers and sick/smokers.

### Quantile Regression reference equation and comparison with GAMLSS

As equations modelled with GAMLSS are complex and cannot be implemented in every spirometer we developed reference equations with quantile regression to increase the implementation possibilities. However, residual analyses revealed a worse fit than for the GAMLSS models for all endpoints (additional information can be found in the supporting information online).

### Sensitivity analysis

A sensitivity analysis was performed for the following variables:

#### Compulsory measurement in children

In 66.5% of all children and adolescents spirometry was done in a compulsory setting. No significant difference was seen when excluding children measured in a volunteer setting.

#### Years of data collection

As the data was collected over a period of 17 years we analysed a linear time trend but did not find any significant difference over time.

#### Body Mass Index (BMI)

Only marginal differences were found when comparing the reference equations for BMI cut-of values of <25, 25–30 and >30 for adults and their equivalents for children [22].

#### Common cold

Reference values for common cold alone were only marginally different than reference values for healthy individuals.

As only marginal differences were found in all sensitivity analysis (data not shown) all individuals and years were included in the final population.

## Discussion

We developed spirometry reference equations for 8–90 year olds from a very large, cross-sectional sample of a Central European population.

“Spirometry reference values are important for the interpretation of individual spirometry measurements and may influence clinical decision making. Most published reference equations use statistical methods that cannot adequately model the complexity of age-dependent lung function [2] and very few span across all ages [3] introducing discontinuities at the transition points with potential clinical implications for individuals with chronic lung diseases.”

One exception are the recently published spirometry reference equations by Stanojevic et al., developed with complex statistical methods for individuals aged 4–80 years of age [8]. As their reference equations were derived from 4 pooled datasets collected in 4 different countries (USA, Canada, UK and Belgium) their reference values can be generalized to other mixed populations with similar ethnic backgrounds. The reference equations we developed are complementary to theirs as they also span from school age to old age and use similar statistical methods. However, they are derived from a single Central European population with homogenous local environmental factors and genetic background and the data was collected using the same instruments and testing procedures throughout the years. Nevertheless, both equations result in similar values [8] (Figure 2: healthy non-smoker). In boys, the peak lung function is reached at the age of 20 years with almost 4.5 l followed by an age-dependent decline to just under 2.75 l at the age of 80 years. In girls, the peak lung function is reached at 19 years with 3.4 l followed by an age-dependent decline to 2 l at the age of 80 years. The decline is initially less steep in the LuftiBus population with 3.2 l at the age of 40 years compared to 3 l in the Stanojevic reference equations.

Spirometry reference equations should be derived from a population as similar to the population from which the patient originates as possible [23]. However, most spirometry reference equations are derived from healthy non-smoking individuals [3] who are generally a small subsample and have higher reference values than the general population [24]. Some authors have therefore included smokers in their reference population when the smoking prevalence was high [25]. The statistical methods we used permitted us to model the entire population while including information on smoking and health status. This allows clinicians to choose which reference values are most appropriate for a given individual. Indeed, although in most situations reference values for healthy-never-smokers will be used, reference values for healthy-smokers might be more appropriate for certain patients when tracking disease progression or assessing effectiveness of therapy over time. By including information on disease the reference equations allow a comparison between healthy and sick individuals. As can be seen in Figure 2 our data confirm that individuals with lung diseases have lower spirometry values than healthy individuals. Even though individuals with common cold where included in the “sick” group, they did not have significantly different reference values than healthy individuals, suggesting as recently published [24] that not all respiratory symptoms need to be accounted for when performing spirometry in patients.

Practically, the reference values according to the GAMLSS model can be calculated by the four functions in Table 2 and the formula found in Figure S3 [10]. To begin with, the values age, sex, and height of a person have to be known. Smoker and sick are for the clinician to decide. If the clinician would like to compare a person to a “healthy-non-smoker” population then “smoker” and “sick” should be set to zero. The values calculated with the Table 2 must then be inserted in the function found in Figure S3 from where the quantiles can be calculated. However, since these are complex algebraic equations, the reference values are best obtained by using the statistical software package R where the function ‘qBCPE’ implemented in the package ‘gamlss’ can be used. R is a free language and environment for statistical computing and graphics that can be downloaded from the following internet site (http://www.r-project.org/). Additionally, upon request, the authors will gladly provide the source code in R, thus facilitating its implementation in spirometry devices.

To allow the reference equations to be implemented in a wide range of spirometers we additionally developed reference equations with quantile regression [12], [26], [27] using the same endpoints (Table S1). A comparison between GAMLSS and quantile regression models was done in Figure S5. However, compared to the GAMLSS models, residual analyses revealed a worse model fit for all endpoints (Figure S1, S4). Therefore, while the GAMLSS reference equations should be used whenever possible as they give the most accurate reference values the quantile regression equations can be implemented as an alternative.

The reference equations for 18–80 years old recently published by Kuster et al [12] are derived from the same data set. However, the two reference equations are not directly comparable. Indeed, we included data spanning from school age to old age thus modelling the growth spurt of puberty and the transition from childhood to adulthood. The equations presented herein therefore expand and complement the reference equations from Kuster at al.

The ATS/ERS task force recommends that reference values be derived from a “representative sample of healthy subjects in a general population”; but, alternatively, can also be derived from a “large group of volunteers, provided that criteria for normal selection and proper distribution of anthropometric characteristics are satisfied” [1]. Although the population visiting the “LuftiBus” consisted mostly of volunteers and was thus possibly motivated by personal health concerns we believe that the “LuftiBus” population can be considered a “large group of volunteers” representative of the Zurich population. First, in the course of the 18 years the “LuftiBus” visited each village of the Zurich County and a similar proportion of the population of each village is represented in the dataset. Second, the age distribution of the “LuftiBus” dataset is similar to the age distribution of the Swiss population apart from an over representation of teenagers [15]. Third, when the “LuftiBus” was leased by schools whole classrooms were tested which allowed us to perform a sensitivity analysis between the children being tested in a compulsory or a voluntary setting. No significant difference was found. Lastly, we excluded all “sick” and “smoking” individuals from our “healthy/non-smoking” reference values, thus reducing possible biases caused by health concerns.

Lung function has been shown to be influenced by various factors such as cohort effect [1], ethnicity [28] or BMI [29]. As only marginal differences were found when performing sensitivity analysis we did not exclude individuals or years tested but rather considered them as part of our “general representative” population.

We developed spirometry reference equations spanning from school age to old age for a Central European population. The equations were derived from a large general population and are intended for every day clinical use as they can be implemented in most clinical settings. Additionally they allow clinicians to choose reference values depending on a given clinical situation.

## Supporting Information

Supporting Information S1 **Results S1; Quantile Regression reference equation and comparison with GAMLSS.**
(DOC)

Table S1 **Quantile regression reference equation.**
(DOCX)

Figure S1 **Residual plots for FEV1 from the GAMLSS model.** Residuals of FEV1 from the GAMLSS model using BCPT are shown: (a) against fitted values of μ (b) against each person (c) kernel density estimate (d) normal QQ plot. The Figures show that the model is adequately fitted as the plots are homogenous, compact, well centred around the zero in the density estimate plot and only about 1484 individuals are not on the QQ-line. GAMLSS: Generalized Additive Models for Location, Scale and Shape. BCPE: Box-Cox power exponential density distribution function. FEV1: forced expiratory volume in one second. μ: mean.
(TIF)

Figure S2 **Worm plot of the residuals of the GAMLSS reference equation for FEV1.** The worm plot shows that the model is well fitted at every age. The top bar shows the 20 age ranges tested (displayed in steps from 6 to 99 years). The 20 corresponding 20 QQ plots (quantile-quantile plots) are probability plots, which is a graphical method for comparing the residuals of the GAMLSS model. They read from bottom left to top right and correspond to the 20 age ranges. GAMLSS: Generalized Additive Models for Location, Scale and Shape. FEV1: forced expiratory volume in one second.
(TIF)

Figure S3 **Formula for calculating quantiles.** Formule taken from Rigby RA, Stasinopoulos DM (2004) Smooth centile curves for skew and kurtotic data modelled using the Box-Cox power exponential distribution. Stat Med 23: 3053–3076.
(TIF)

Figure S4 **Residual plots for FEV1 for quantile regression.** Residuals from the quantile regression model for the 50th and the 5th quantile are shown. (a) against fitted values of μ (b) against each person (c) kernel density estimate (d) normal QQ plot. The residuals show a slight skewed distribution which is accentuated in the 5th quantile. This can be seen by the plots being less centred and less compact, having individuals at −4 but non at +4 in the density estimate plot and having less individuals on the QQ-line. FEV1: forced expiratory volume in one second. μ: mean.
(TIF)

Figure S5 **Comparison between the GAMLSS and Quantile Regression reference equations.** The lung function parameter FEV1 is compared between the GAMLSS and the Quantile Regression model between the ages of 8–90 years old. For this comparison only healthy non-smoking men of 175 cm and women of 165 cm were included. The 5^th^ quantile indicates the lower limit of normal for each group. GAMLSS: Generalized Additive Models for Location, Scale and Shape. Quantreg: quantile regression.
(TIF)
