# Supplementary material for: Spirometry Reference Equations for Central European Populations from School Age to Old Age
Source: PLoS One. 2013 Jan 8;8(1):e52619. doi: 10.1371/journal.pone.0052619 (PMC3540072; doi:10.1371/journal.pone.0052619)
Supplement: Supporting Information S1 — Results S1; Quantile Regression reference equation and comparison with GAMLSS. (DOC) [file pone.0052619.s001.doc]

**RESULTS S1**

**Quantile Regression reference equation and comparison with GAMLSS**

As equations modelled with GAMLSS are complex and cannot be implemented in every spirometer we developed reference equations with quantile regression to increase the implementation possibilities. Within the framework of the quantile regression we estimated the 5% and 50% quantiles for all spirometry values. The reference values (5th quantile) can be calculated by the function in Table S1. Graphically based residual analyses were done in order to identify model inadequacies (Figure S4). However, residual analyses revealed a worse fit than for the GAMLSS models for all endpoints. A comparison between GAMLSS and quantile regression models was done by comparing regression coefficients of the mean of the GAMLSS model with the regression coefficients of the quantile regression models. In Figure S5 the estimated values for the 50th and 5th quantiles for each age can be seen. As the other covariates were fixed, only healthy non-smoking men of 175 cm and women of 165 cm were included. Although the GAMLSS model shows a much better fit (Figure S1, S2) than the quantile regression fit (Figure S4) the estimated values obtained by both methods were similar (Figure S5).
