# Supplementary material for: Spirometry Reference Equations for Central European Populations from School Age to Old Age
Source: PLoS One. 2013 Jan 8;8(1):e52619. doi: 10.1371/journal.pone.0052619 (PMC3540072; doi:10.1371/journal.pone.0052619)
Supplement: Table S1 — Quantile regression reference equation. (DOCX) [file pone.0052619.s002.docx]

**Table S1: Quantile regression reference equation**

| **Reference equation for the 50^th^ quantile** | | | | | | | |
| --- | --- | --- | --- | --- | --- | --- | --- |
| **g_QR_ = β0 +β1*age + β2* √ ((age – α1)² +γ1²) + β3* √ ((age – α2)² +γ2²) + β4*sex + β5*height + β6*smoker + β7*sick + β8* age * sex + β9* √ ((age – α1)² +γ1²) * sex + β10* √ ((age – α2)² +γ2²) * sex + β11* height * sex** | | | | | | | |
|  | **FEV1** | **FVC** | **FEV1/FVC** | **PEF** | **MEF25** | **MEF50** | **MEF75** |
| **Regression parameters (β):** | | | | | | | |
| **β0** | **-3.757** | **-5.705** | **105.338** | **-0.113** | **0.799** | **-0.493** | **-2.671** |
| **β1** | **0.049** | **0.029** | **0.453** | **0.152** | **0.102** | **0.110** | **0.064** |
| **β2** | **-0.074** | **-0.047** | **-0.778** | **-0.211** | **-0.154** | **-0.159** | **-0.120** |
| **β3** | **-0.013** | **-0.022** | **0.162** | **-0.096** | **-0.065** | **-0.016** | **0.046** |
| **β4** | **1.738** | **2.384** | **3.346** | **0.750** | **0.803** | **1.694** | **0.783** |
| **β5** | **0.043** | **0.061** | **-0.146** | **0.073** | **0.049** | **0.028** | **0.012** |
| **β6** | **-0.046** | **0.013** | **-1.450** | **-0.147** | **-0.062** | **-0.172** | **-0.122** |
| **β7** | **-0.092** | **-0.065** | **-0.942** | **-0.188** | **-0.360** | **-0.297** | **-0.086** |
| **β8** | **-0.029** | **-0.020** | **-0.104** | **-0.109** | **-0.077** | **-0.063** | **-0.033** |
| **β9** | **0.033** | **0.026** | **0.010** | **0.123** | **0.094** | **0.074** | **0.034** |
| **β10** | **0.002** | **-0.001** | **0.097** | **0.041** | **0.033** | **0.005** | **0.003** |
| **β11** | **-0.011** | **-0.016** | **-0.004** | **-0.017** | **-0.013** | **-0.008** | **-0.003** |
| **Changepoints (α)** | | | | | | | |
| **α1** | **18.735** | **21.359** | **15.989** | **19.838** | **19.373** | **17.176** | **16.573** |
| **α2** | **37.027** | **35.958** | **25.750** | **53.841** | **57.885** | **57.004** | **12.000** |
| **Transition smoothness (γ)** | | | | | | | |
| **γ1** | **0.864** | **0.230** | **0.329** | **1.010** | **1.257** | **0.821** | **0.206** |
| **γ2** | **13.578** | **12.185** | **5.537** | **29.938** | **17.258** | **1.012** | **49.328** |
| **Reference equation for the 5^th^ quantile** | | | | | | | |
| **g_QR_ = β0 +β1*age + β2* √ ((age – α1)² +γ1²) + β3* √ ((age – α2)² +γ2²) + β4*sex + β5*height + β6*smoker + β7*sick + β8* age * sex + β9* √ ((age – α1)² +γ1²) * sex + β10* √ ((age – α2)² +γ2²) * sex + β11* height * sex** | | | | | | | |
|  | **FEV1** | **FVC** | **FEV1/FVC** | **PEF** | **MEF25** | **MEF50** | **MEF75** |
| **Regression parameters (β)** | | | | | | | |
| **β0** | **-3.335** | **-4.901** | **102.764** | **-3.120** | **0.041** | **-0.929** | **-12.466** |
| **β1** | **0.032** | **0.024** | **0.338** | **0.102** | **0.080** | **0.064** | **0.058** |
| **β2** | **-0.065** | **-0.052** | **-0.722** | **-0.145** | **-0.142** | **-0.104** | **-0.075** |
| **β3** | **-0.012** | **-0.017** | **-0.156** | **-0.076** | **-0.045** | **-0.009** | **0.075** |
| **β4** | **1.310** | **2.129** | **2.612** | **2.056** | **0.866** | **1.359** | **1.972** |
| **β5** | **0.038** | **0.052** | **-0.139** | **0.067** | **0.035** | **0.021** | **0.008** |
| **β6** | **-0.094** | **-0.012** | **-2.734** | **-0.283** | **-0.281** | **-0.257** | **-0.085** |
| **β7** | **-0.224** | **-0.131** | **-4.056** | **-0.338** | **-0.764** | **-0.429** | **-0.095** |
| **β8** | **-0.019** | **-0.022** | **-0.115** | **-0.077** | **-0.046** | **-0.027** | **-0.022** |
| **β9** | **0.028** | **0.032** | **0.200** | **0.090** | **0.067** | **0.033** | **0.023** |
| **β10** | **0.004** | **0.001** | **0.095** | **0.025** | **0.021** | **0.004** | **-0.008** |
| **β11** | **-0.009** | **-0.014** | **-0.020** | **-0.017** | **-0.008** | **-0.007** | **-0.002** |
| **Changepoints (α)** | | | | | | | |
| **α1** | **19.240** | **21.023** | **15.000** | **19.224** | **17.777** | **17.006** | **17.071** |
| **α2** | **43.283** | **41.375** | **57.648** | **42.908** | **50.031** | **43.955** | **57.324** |
| **Transition smoothness (γ)** | | | | | | | |
| **γ1** | **0.532** | **0.000** | **0.000** | **0.000** | **1.139** | **0.000** | **0.116** |
| **γ2** | **5.778** | **8.201** | **0.100** | **19.986** | **6.869** | **2.077** | **150.000** |

The variables are coded as followed:

Age: years; height: cm, sex: male = 0, female = 1; smoker: non-smoker = 0; smoker = 1*; sick: healthy = 0; sick = 1*

*definition of smoker and sick can be found in the methods section of the paper.

**Note:** to compare a patient with a “healthy-non-smoker” population, “smoker” and “sick” must be set to “0” even if the patient is a smoker and has a pulmonary pathology.
