# Supplementary figures and images for: Spirometry Reference Equations for Central European Populations from School Age to Old Age
Source: PLoS One. 2013 Jan 8;8(1):e52619. doi: 10.1371/journal.pone.0052619 (PMC3540072; doi:10.1371/journal.pone.0052619)

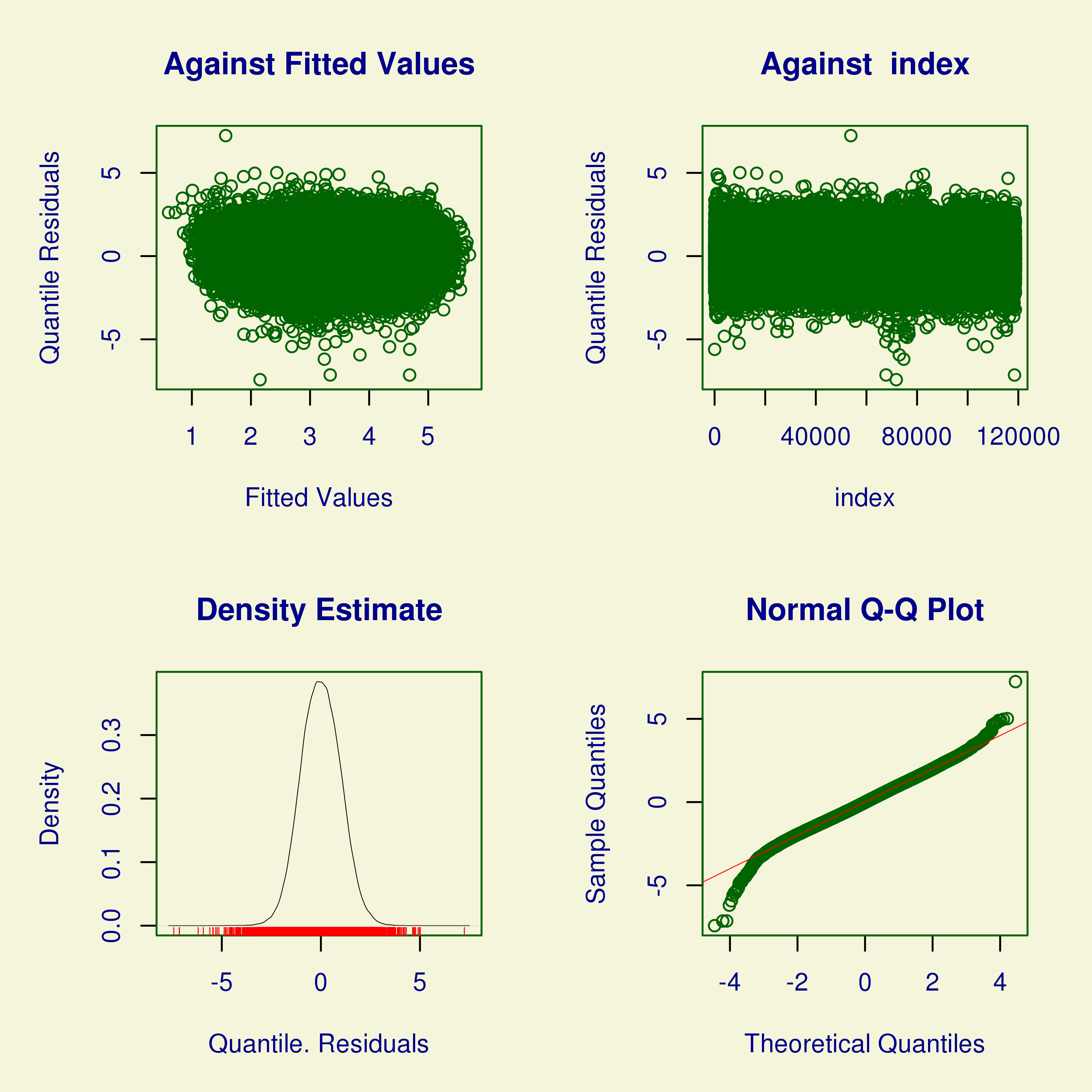

Supplement: Figure S1 — Residual plots for FEV1 from the GAMLSS model. Residuals of FEV1 from the GAMLSS model using BCPT are shown: (a) against fitted values of μ (b) against each person (c) kernel density estimate (d) normal QQ plot. The Figures show that the model is adequately fitted as the plots are homogenous, compact, well centred around the zero in the density estimate plot and only about 1484 individuals are not on the QQ-line. GAMLSS: Generalized Additive Models for Location, Scale and Shape. BCPE: Box-Cox power exponential density distribution function. FEV1: forced expiratory volume in one second. μ: mean. (TIF) [file pone.0052619.s003.tif]

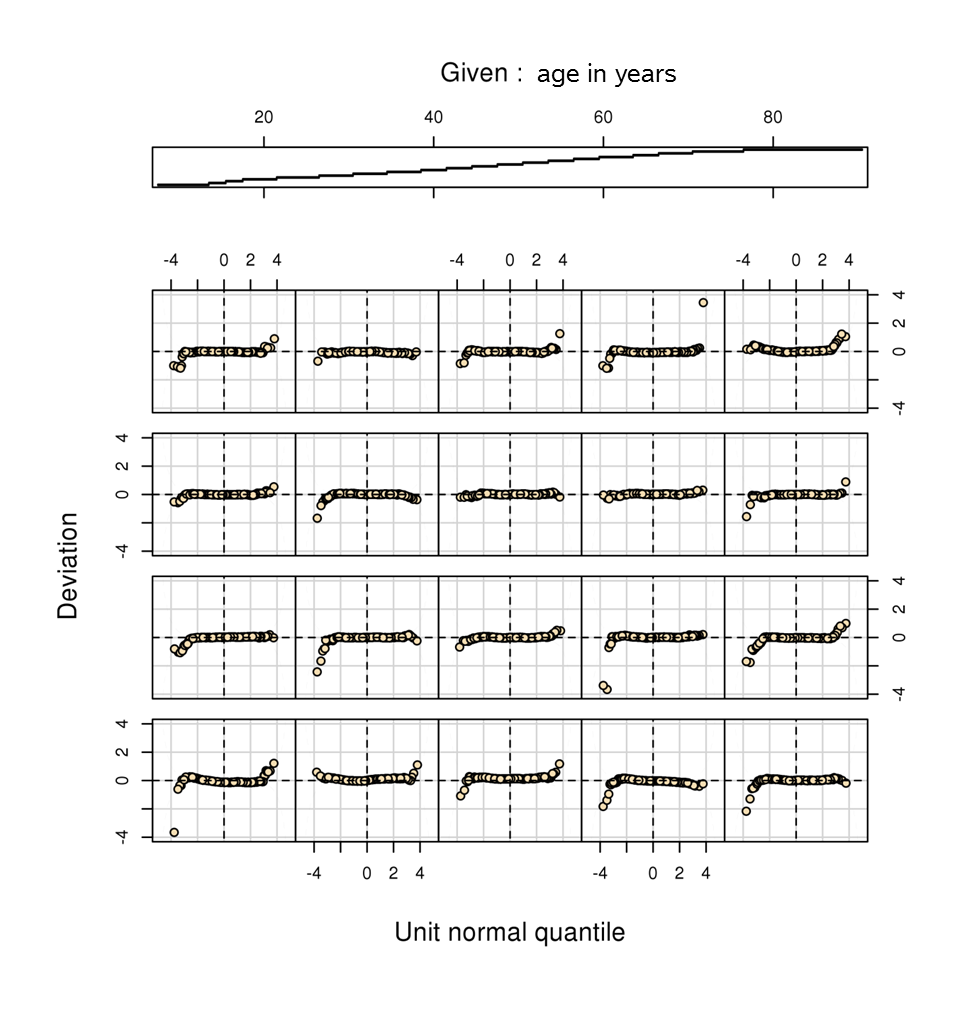

Supplement: Figure S2 — Worm plot of the residuals of the GAMLSS reference equation for FEV1. The worm plot shows that the model is well fitted at every age. The top bar shows the 20 age ranges tested (displayed in steps from 6 to 99 years). The 20 corresponding 20 QQ plots (quantile-quantile plots) are probability plots, which is a graphical method for comparing the residuals of the GAMLSS model. They read from bottom left to top right and correspond to the 20 age ranges. GAMLSS: Generalized Additive Models for Location, Scale and Shape. FEV1: forced expiratory volume in one second. (TIF) [file pone.0052619.s004.tif]

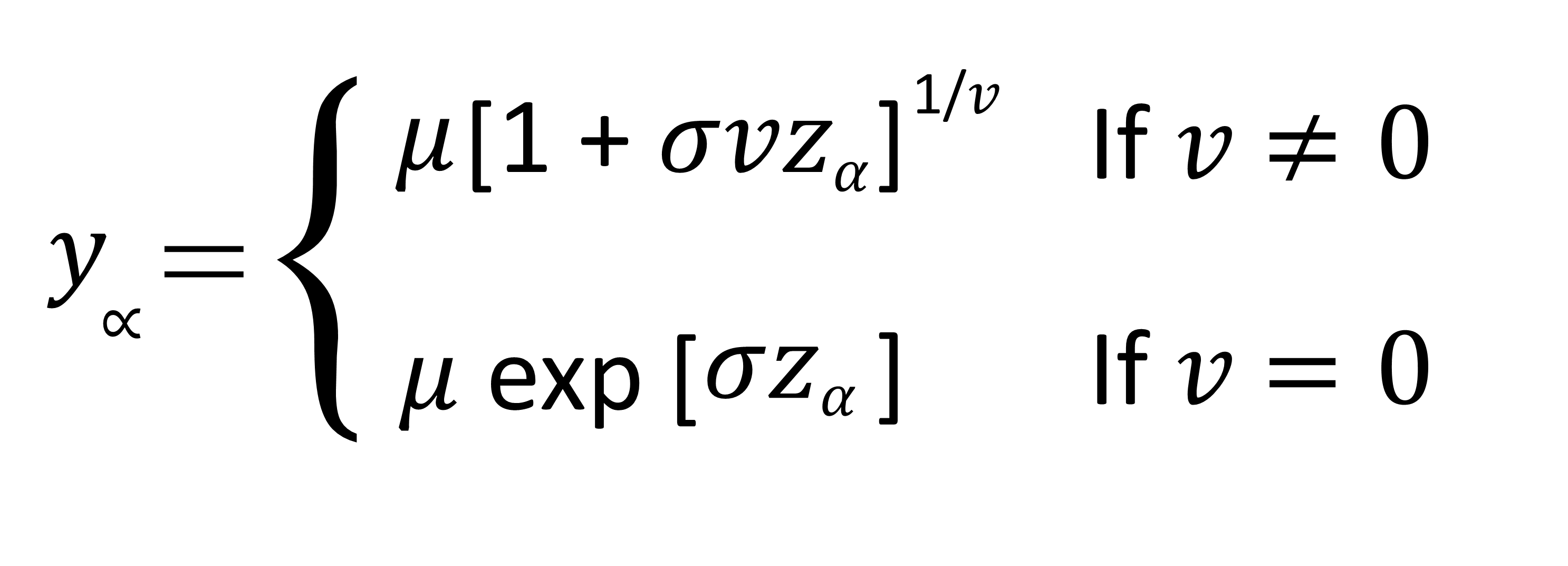

Supplement: Figure S3 — Formula for calculating quantiles. Formule taken from Rigby RA, Stasinopoulos DM (2004) Smooth centile curves for skew and kurtotic data modelled using the Box-Cox power exponential distribution. Stat Med 23: 3053–3076. (TIF) [file pone.0052619.s005.tif]

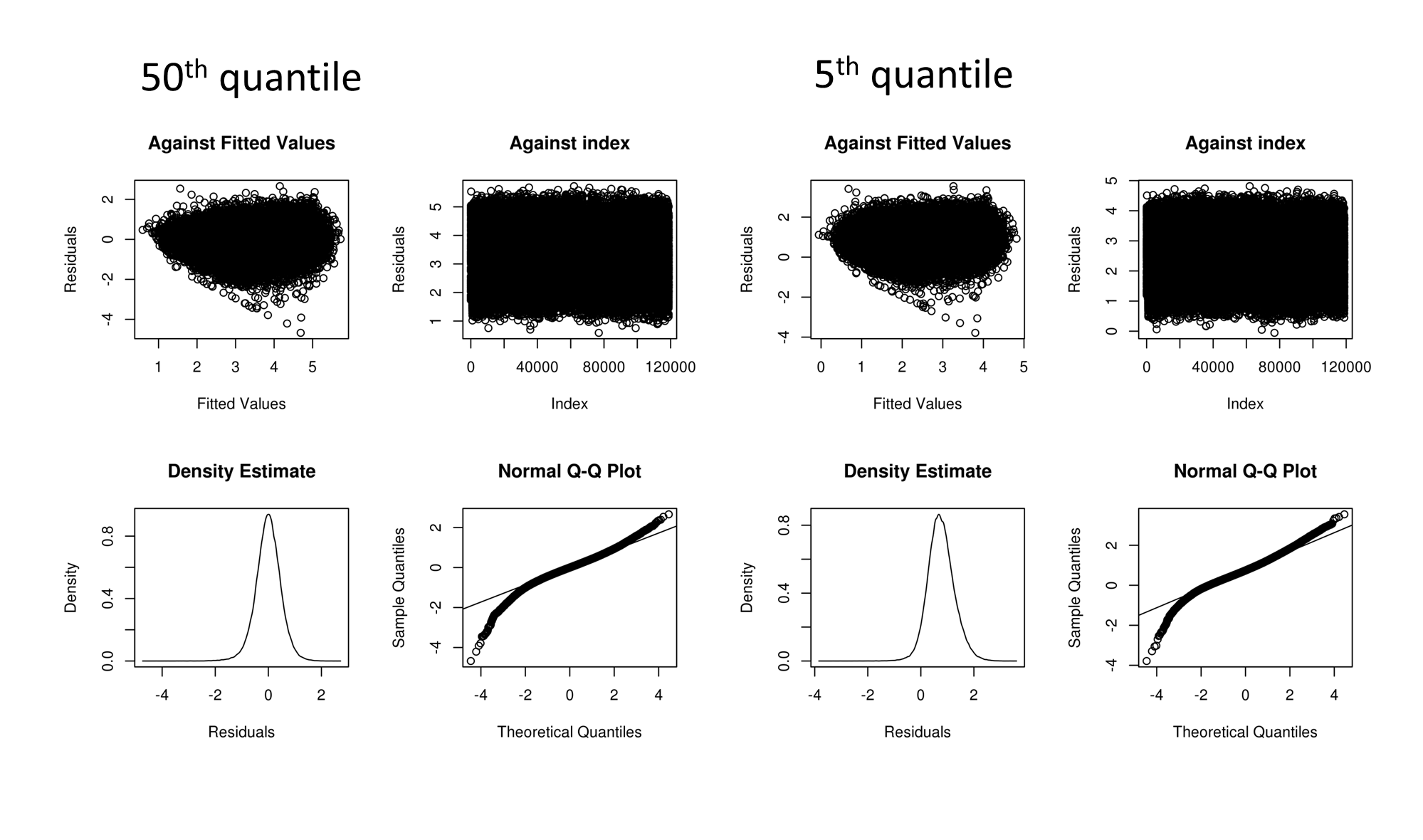

Supplement: Figure S4 — Residual plots for FEV1 for quantile regression. Residuals from the quantile regression model for the 50th and the 5th quantile are shown. (a) against fitted values of μ (b) against each person (c) kernel density estimate (d) normal QQ plot. The residuals show a slight skewed distribution which is accentuated in the 5th quantile. This can be seen by the plots being less centred and less compact, having individuals at −4 but non at +4 in the density estimate plot and having less individuals on the QQ-line. FEV1: forced expiratory volume in one second. μ: mean. (TIF) [file pone.0052619.s006.tif]

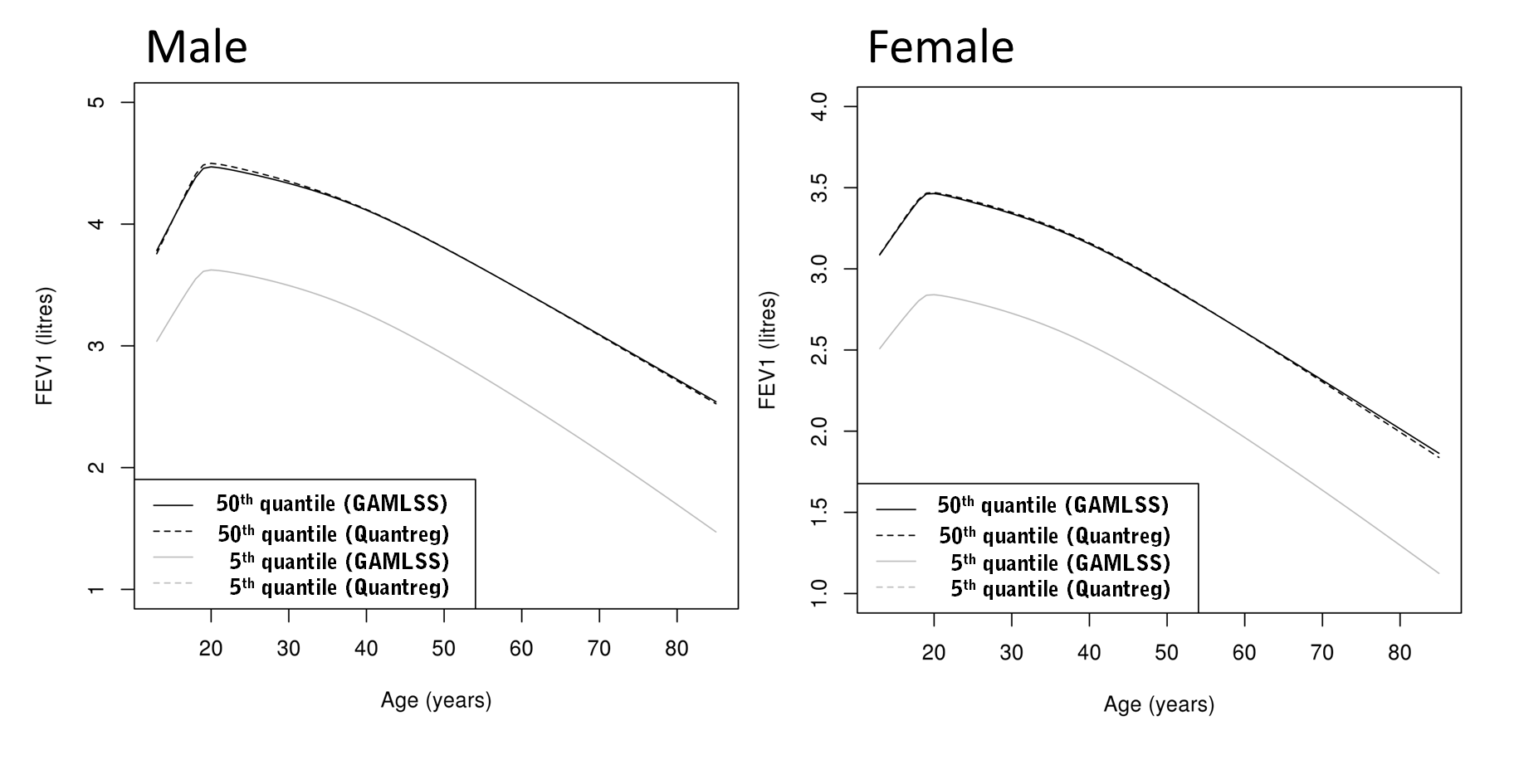

Supplement: Figure S5 — Comparison between the GAMLSS and Quantile Regression reference equations. The lung function parameter FEV1 is compared between the GAMLSS and the Quantile Regression model between the ages of 8–90 years old. For this comparison only healthy non-smoking men of 175 cm and women of 165 cm were included. The 5th quantile indicates the lower limit of normal for each group. GAMLSS: Generalized Additive Models for Location, Scale and Shape. Quantreg: quantile regression. (TIF) [file pone.0052619.s007.tif]
